# Copy Number Variation Analysis of Matched Ovarian Primary Tumors and Peritoneal Metastasis

**DOI:** 10.1371/journal.pone.0028561

**Published:** 2011-12-14

**Authors:** Joel A. Malek, Eliane Mery, Yasmin A. Mahmoud, Eman K. Al-Azwani, Laurence Roger, Ruby Huang, Eva Jouve, Raphael Lis, Jean-Paul Thiery, Denis Querleu, Arash Rafii

**Affiliations:** 1 Genomics Core, Weill Cornell Medical College in Qatar, Education City, Qatar Foundation, Doha, Qatar; 2 Institut Claudius Regaud, Toulouse, France; 3 Department of Obstetrics and Gynecology and Cancer Science Institute of Singapore, National University of Singapore, Singapore, Singapore; 4 Stem Cell and Microenvironment Laboratory, Weill Cornell Medical College in Qatar, Education City, Qatar Foundation, Doha, Qatar; 5 Institute of Molecular and Cell Biology, A*STAR, Singapore, Singapore; 6 McGill University, Montreal, Canada; 7 Department of Genetic Medicine, Weill Cornell Medical College, New York, New York, United States of America; Barts & The London School of Medicine and Dentistry, Queen Mary University of London, United Kingdom

## Abstract

Ovarian cancer is the most deadly gynecological cancer. The high rate of mortality is due to the large tumor burden with extensive metastatic lesion of the abdominal cavity. Despite initial chemosensitivity and improved surgical procedures, abdominal recurrence remains an issue and results in patients' poor prognosis. Transcriptomic and genetic studies have revealed significant genome pathologies in the primary tumors and yielded important information regarding carcinogenesis. There are, however, few studies on genetic alterations and their consequences in peritoneal metastatic tumors when compared to their matched ovarian primary tumors. We used high-density SNP arrays to investigate copy number variations in matched primary and metastatic ovarian cancer from 9 patients. Here we show that copy number variations acquired by ovarian tumors are significantly different between matched primary and metastatic tumors and these are likely due to different functional requirements. We show that these copy number variations clearly differentially affect specific pathways including the JAK/STAT and cytokine signaling pathways. While many have shown complex involvement of cytokines in the ovarian cancer environment we provide evidence that ovarian tumors have specific copy number variation differences in many of these genes.

## Introduction

Epithelial Ovarian carcinoma (EOC) is the sixth most common malignancy in woman and the leading cause of death from gynecological cancer in the world [1]. The poor overall survival (20 to 30% at 5 years) is due to the large tumor burden with extensive metastatic lesions of the peritoneal cavity. Despite initial chemosensitivity and improved surgical procedures abdominal recurrence remain an issue and results in patients' poor prognosis. Therefore it is critical to understand the molecular pathways underlying peritoneal metastasis in order to define new therapeutic strategies [2].

Efforts have been made to delineate gene expression signatures for prognostic predictions as well as chemotherapeutic responses [3]–[6]. These studies have attempted to provide gene predictors on disease outcome, however, the robustness and reproducibility of these genes lists across different patient populations have not yet been clearly established or translated to clinical practice [7].

The complex cytogenetic alterations of ovarian carcinoma and the lack of high-resolution technologies have hindered the identification of specific genes involved in the metastatic process. Using low-resolution platforms, wide-spread copy number changes of 7 amplicons (CCNE1, Notch3, HBXAP/Rsf-1, AKT2, PIK3CA and chr12p13) in high-grade tumors were identified while a relatively flat and quiet chromosomal landscape was found in low-grade tumors [8]. Recently, analysis performed by the TCGA and other groups with much higher resolution platforms have shown numerous and frequent micro-deletions and amplifications across the genome, with genes CCNE1, RB1, MYC, MECOM and FGFR1 highlighted among others [9], [10]. While recent studies of high number of patients have led to the precise characterization of the genetic alterations in serous ovarian carcinoma [11], there has been little effort, to our knowledge, to understand the dynamics of large scale genetic modification differences between the primary lesions and the peritoneal metastasis. In a study on loss of heterozygosity, Khalique and colleagues compared primary and metastatic ovarian tumors using 22 microsatellite markers in 22 patient samples [12]. Despite the low resolution resulting in a lack of functional analysis their overall findings on tumor progression agree with ours presented here.

Therefore we hypothesize that a prospective collection of homogenous primary and metastatic lesions from patients with advanced ovarian carcinoma would allow a comprehensive view of genetic modification and have the potential to define important pathways for the occurrence of peritoneal metastasis in serous papillary ovarian carcinoma.

## Results

We identified 9 patients with matched ovarian and peritoneal metastatic tumors (Table 1). All primary tumors were grade 3 papillary serous tumors stage IIIc, and all patients had primary upfront debulking surgery. One biopsy was performed from the primary tumor, and one biopsy was performed from a peritoneal metastatic lesion without the underlying peritoneal stroma.

**Table 1:** Patient Information used in this study.

Age	61 +/− 7
Histology (9 patients)	Papillary-serous adenocarcinoma
Grade (9 patients)	3
Stage	
IIIC	8
IV (pleural)	1 (patient 07c3714)
Adjuvant treatment	Carboplatin and taxol (6 cycles)

We used the Affymetrix SNP 6.0 chip to detect regions with significant copy number variations (CNV) with respect to either a HapMap control set or the matched primary tumors. For validation, we selected 14 regions for quantitative-PCR validation of peritoneal metastasis versus primary tumor copy number. The regions included 3 controls shown to not be within CNVs in the patient's studied here, and an additional 11 regions (Table S1) within 5 genes showing CNV among the patients. We had sufficient DNA from 16 of the 18 tumors investigated by array (DNA from patient OV07-3 was insufficient) for qPCR validation. Our results (Table S2) show that 83% of regions agreed between the qPCR and Array data. We detected 7.5% false negatives (missed CNVs) and 10% false positives. These data are conservative in assuming qPCR is always correct. The results here agree favorably with previous findings [13], [14] for the frequency of qPCR agreement with data from Affymetrix SNP 6.0 data analyzed with PARTEK software.

### Primary and metastatic comparison to normal

**Figure 1:**
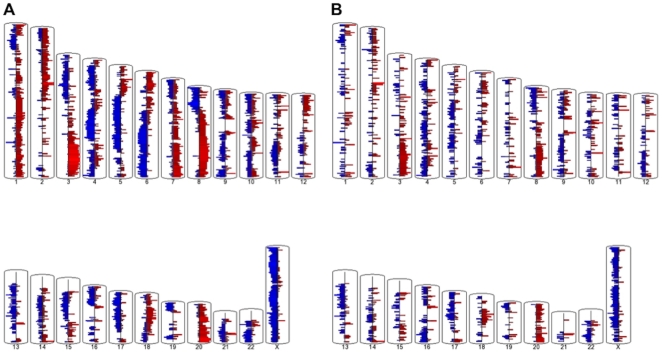
Ovarian tumor copy numbers in genomic DNA compared to a normal baseline. A) Primary tumors. B) Peritoneal metastasis. Amplifications are in red and deletions in blue.

We first compared genomic DNA from primary and metastatic lesions with a dataset of normal tissues provided by the HapMap project. This should yield cancer specific amplifications and deletions when compared to normal tissue (Figure 1). Only regions amplified or deleted in at least 3 samples were documented. In individual patients, segments of amplification and deletion could be quite long, however, when compared among multiple patients the boundaries of CNVs were tightened making the average CNV segment ∼200 kb. There were 8681 segments, spanning 2.1 Gb of sequence, detected as CNVs in at least 3 patients in the primary tumors (Table S3). 4176 were amplifications spanning 957 Mb with average segment size of 230 kb. 4257 were deletions spanning 1152 Mb with average segment size of 270 kb. On average an individual patient had 2445 amplified segments spanning 530 Mb and 2412 deleted segments spanning 651 Mb. There were 5878 segments detected as CNVs in at least 3 patients in the peritoneal metastasis samples (Table S4). 2445 were amplifications spanning 364 Mb with average segment size of 149 kb. 3366 were deletions spanning 621 Mb with average segment size of 184 kb. On average an individual patient had 1289 amplified segments spanning 170 Mb and 1542 deleted segments spanning 290 Mb in the peritoneal metastasis. Encouragingly, the most frequent amplifications and deletions agreed with previous published studies [10]. These included amplifications in 3q, 6p, 8q, 12p and 20, and deletions in 4q, 5q, 6q, 8p, 16p, 17, 22, and X among others (Table S3, Table S4). Genes in CNVs, both shared and tumor specific, were documented in Table S5.

While our data agrees well with previous ovarian CNV studies [10], little has been done to characterize functional pathways affected by these consistently amplified and deleted regions. Functional analysis by DAVID [15] of genes within regions amplified in both primary and metastatic regions revealed enrichment of genes involved in the JAK/STAT signaling pathway (29 genes, Benjamini-Hochberg (BH) score 0.0067) and Cytokine-Cytokine Receptor pathway (38 genes, BH score 0.06) among others. Deletions were enriched for genes involved in Apoptosis (28 genes, BH 0.092), MAPK signaling (66 genes, BH score 0.099) among others. Deleted apoptosis related genes included p53, ATM, Bcl-2, NF-kB, IKK, CASP3 and CASP6 among others. It is clear from our analysis that the metastasis has fewer specific copy number changes (17× fewer specific amplifications and 3× fewer deletions on average) when compared with the primary tumor (Table S5). A similar trend was observed in breast cancer where primary tumors had on average 20% more changes than their metastatic counterparts [16]. This may be due to a microenvironment pressuring the metastatic tumor to maintain certain pathways. The disproportionate number of primary tumor specific amplifications may suggest a concerted requirement, post-metastatic spread, to increase gene copy number in specific genes for tumor maintenance. It might also suggest that the metastatic event occurs quite early in the occurrence of the disease with the metastatic clones being less prone to amplifications and deletions in their new microenvironment and this is discussed below.

For corroboration of pathways identified in the CNV analysis, we searched gene expression data of the same samples (Malek *et al.*, in preparation) for genes differentially expressed between primary and metastatic tumors. Functional analysis of gene expression data also revealed enrichment of differentially expressed genes in the cytokine/receptor interaction pathway and in the JAK-STAT signaling pathway. These findings are not unexpected as the underlying CNVs likely affect gene expression.

To our knowledge the identification of significant enrichment of JAK-STAT signaling and cytokine/receptor genes within ovarian CNVs is novel. Given that both primary and metastatic tumor CNVs showed highly significant enrichment of these genes, and because gene expression data showed genes in these pathways to be differentially expressed between primary and metastatic tumors, we chose to focus our functional analysis on these pathways. Groups of amplifications and deletions (shared by both primary and metastatic, primary specific, metastatic specific, and peritoneum compared directly to primary) were mapped to the JAK/STAT signaling pathway (Table 2). It is clear from this analysis that multiple genes in the JAK/STAT pathway are affected by CNVs and indeed genes within the same category (for example Interleukins) may be both amplified and deleted in the same comparison. These amplifications and deletions in the same categories are likely coordinated. For example, 5 patients had deletions in the TYK2 gene (a JAK protein), yet 3 of these (patients OV07-1, OV07-4 and OV08-3) were the only 3 patients amplified in the JAK2 gene. This may be an indication of constraints on the balancing of loss and gains in this pathway. Dense copy number variations from multiple comparisons were observed in the cytokine/chemokine signaling pathways and these gene families were analyzed with more detail.

**Table 2:** Copy Number Variations in the KEGG JAK/STAT pathway gene families.

	Shared		Peri Specific		Prim Specific		Peri V Prim	
Gene Symbol	Amp	Del	Amp	Del	Amp	Del	Amp	Del
**EPO**	X		X		X	X	X	X
**IFN/IL10**	X				X		X	X
**IL2/3**	X	X				X	X	X
**IL6**	X	X			X	X	X	X
**CytokineR**	X	X			X	X	X	X
**JAK**	X	X			X		X	X
**Cbl**								
**STAM**							X	
**SHP1**		X			X			X
**SHP2**		X			X			X
**GRB**								
**SOS**					X	X		X
**PI3K**	X	X			X	X	X	
**AKT**			X	X	X			X
**STAT**		X					X	X
**PIAS**		X	X		X		X	X
**SOCS**		X			X	X		X
**IFNalpha/P48**								
**CBP**		X					X	
**Pim-1**								
**CIS**						X	X	
**c-Myc**	X							X
**CycD**					X			X
**BclXL**	X							X
**Spred**					X	X		X
**Sprouty**		X				X		

**Figure 2:**
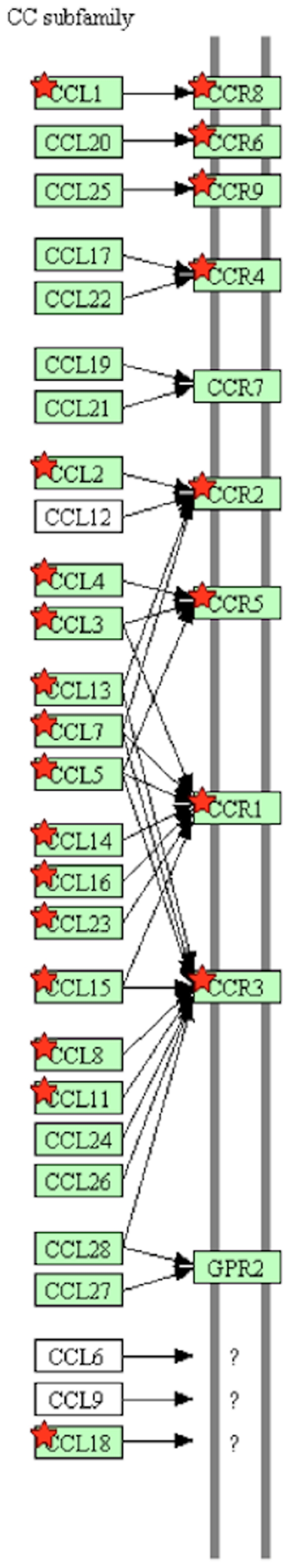
The CC chemokine subfamily deletions in primary but not metastatic tumors. Large numbers of CC subfamily chemokines and there receptors were deleted (starred red) in the primary tumor but not in matched peritoneal metastasis.

We documented all cytokine/chemokine signaling pathway related genes in CNVs and observed trends specific to primary and metastatic tumors (Table S6). Specifically, large numbers of cytokines of the CC subfamily (and corresponding receptors) were specifically deleted in the primary tumor and not in the metastasis (Figure 2, Table 3). This is of interest as it has been shown previously that primary tumors are deficient in CC chemokine receptor [17]. For example we observed a deletion in the CCL2 (LOH in 17q) gene which has been observed to delete in 70% of primary ovarian tumors [18] however this extremely common primary tumor deletion was not observed in the peritoneal metastasis. This lack of deletion was irrespective of age of the metastasis (discussed below). The fact that the metastasis does not delete these genes shows a clear difference between the metastasis and the primary tumors and may likely be due to functional constraints required for the spread and maintenance in a different microenvironment. The role of the CC chemokine family in cancer progression has been widely described in the literature [19]. We observed primary tumor deletion in multiple CC chemokine genes (ligand or receptors) in all but one patient (OV08-2) suggesting this pathway is altered with high frequency (Table 3). Interestingly, patient OV08-2 was the only patient with refractory cancer in our cohort. Whether the complete absence of CC chemokine deletions in this patient's primary tumor is related to this refractory state will require further investigation of similar patients.

**Table 3:** CC family Cytokines and Receptors deleted in primary but not metastatic tumors.

Chr	Seg Start	Seg End	band	Gene	OV06-1	OV07-1	OV07-2	OV07-3	OV07-4	OV07-5	OV08-1	OV08-2	OV08-3
**CCL Deletions**													
2	228282576	228865596	2q36.3	CCL20		X				X	X		X
17	30534992	33419581	17q11.2-17q12	CCL1, CCL11, CCL13, CCL2, CCL7, CCL8			X	X		X			X
17	33419581	34436129	17q12	CCL14, CCL14-CCL15, CCL15, CCL16, CCL18, CCL23, CCL3, CCL4, CCL5			X	X		X			X
17	34436918	34475913	17q12	CCL4			X	X		X			X
17	34475913	34539497	17q12	CCL3L1, CCL3L3, CCL4L1			X	X		X	X		X
17	34539497	34722976	17q12	CCL3L1, CCL3L3, CCL4L1, CCL4L2			X	X		X	X		X
19	8024718	8331609	19p13.2	CCL25	X	X			X	X			X
**CCR Deletions**													
3	32955208	33124328	3p22.3	CCR4	X		X				X		X
3	38997402	39507653	3p22.2-3p22.1	CCR8	X		X						X
3	45325066	47052314	3p21.31	CCR1, CCR2, CCR3, CCR5, CCR9, CCRL2	X	X	X						
6	166770236	169503025	6q27	CCR6	X	X	X	X	X				
17	40305133	41203435	17q21.2-17q21.31	CCR10		X	X	X		X			X

Chr: chromosome, seg start/end: segment start and end.

Likewise, large numbers of the CXC chemokine subfamily were amplified in primary but not metastatic lesions (Table S6), however this showed much more variability than the CC subfamily. Between 3 and 4 patients were amplified while between 2 to 3 were deleted in the same genes. In the matched metastasis only 1 to 2 patients showed CNVs in these genes and were not reported. This lack of consistency for the CXC subfamily CNVs results in failing to meet the threshold set for reporting. CXCL12, which stimulates ovarian cancer cell invasion, was amplified and has been shown to be expressed in ovarian cancer but not normal ovaries or ovarian surface epithelium [20]. However, the lack of consistency in the CXC subfamily CNVs may indicate these are either secondary genes to a more critical pathway or that there may be coordination of amplification and deletion between these and other genes.

We further searched gene expression data of the same samples (Malek *et al.*, in preparation) for genes contained within a CNV and that had significant gene expression differences between primary and metastatic tumors. Of 9 differentially expressed chemokine pathway genes, 6 had the expected gene expression trend based on the CNV. Specifically, CXCR6, CCR2, CCR4, IL2Ra were both deleted and had lower gene expression in the primary versus metastatic tumors. Conversely, CCL28 and VEGF-A were both amplified and had higher gene expression in the primary versus metastatic tumors.

### Matched Metastatic/Primary Tumor CNV analysis

**Figure 3:**
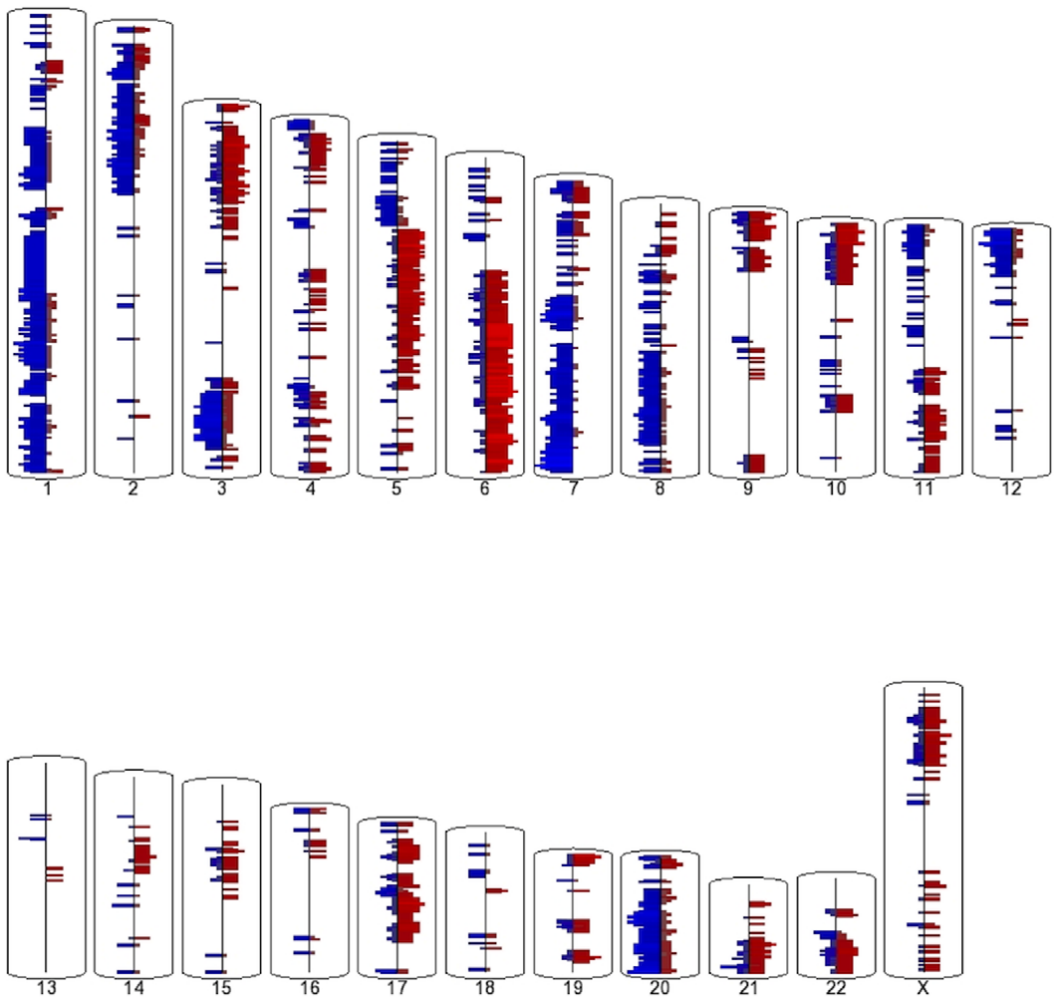
Peritoneal metastasis copy number variation compared to matched ovarian primary tumors. Amplifications are in red and deletions in blue. This comparison highlights differences between matched patient samples and helps identify regions of ongoing copy number change.

While both primary and metastatic lesion CNVs showed significant overlap with previous studies, large regions were different between the two types (Figure 3). These differences may indicate selective pressure based on different microenvironments, selective pressure based on the requirement of metastasizing, or simply the difference between two clonal populations within the primary tumor prior to the event of metastasis. These differences were highlighted even more when matched primary and metastatic lesions were directly compared to each other for all 9 patients to reveal possible metastatic specific trends (Figure 3). There were 2355 segments amplified and 2734 segments deleted in at least 3 patients in the peritoneal metastasis compared to the primary tumor (Table S7). These spanned 562 Mb with average segment size 239 kb and 590 Mb span with average segment size of 216 kb respectively.

**Figure 4:**
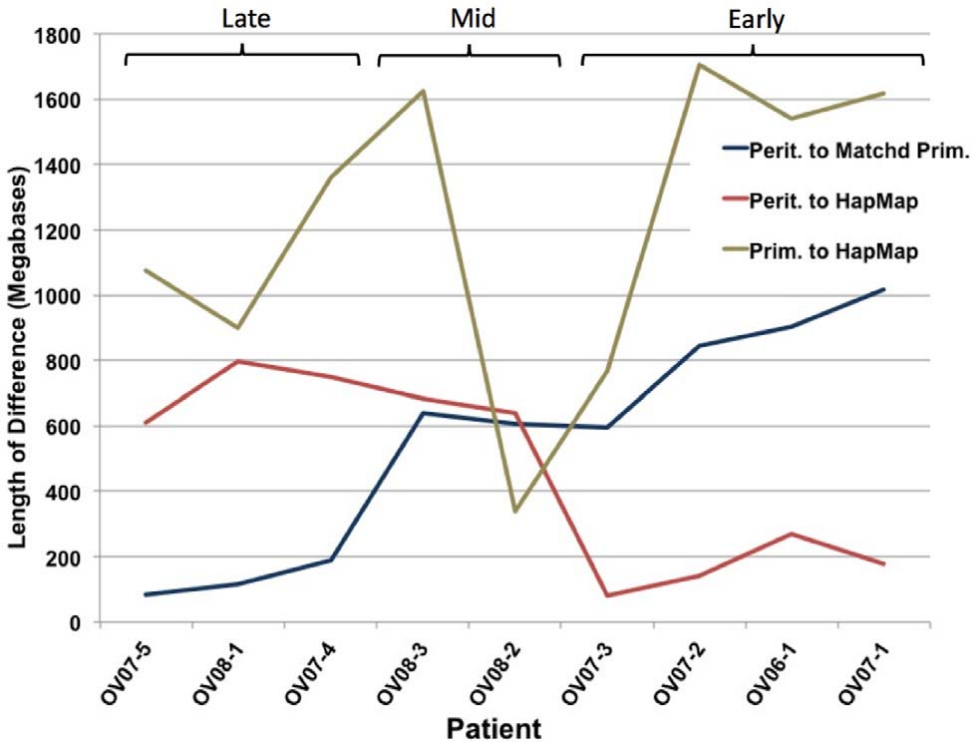
Lengths of copy number variations shared among tumor types differ among patients. Lengths of shared copy number variations between peritoneal (perit.) metastasis and matched primary (prim.) tumors were plotted for each patient. Peritoneal metastases that do not differ much from their primary tumors tend to have large numbers of differences to the HapMap (normal) baseline and likely metastasized only recently. We only observed one patient (OV08-2 with fewer primary tumor CNVs than the peritoneal metastasis). These data suggest groups of early, mid and late metastatic groups separated by amount of shared CNVs between the tumor types.

We observed significant differences among patients in the number of shared CNVs between matched primary and metastatic lesions (Figure 4). This information may allow a better understanding of tumor progression timelines, as more recently metastasized tumors are likely to share more CNVs in common. Likewise early metastatic events would allow longer divergence time between primary and metastatic tumors and would result in large numbers of CNV differences. Three patients showed very few differences between the primary and peritoneal metastasis and large numbers of shared differences to the HapMap baseline suggesting that the metastatic event was recent (late in tumor progression). Four patients had large numbers of differences between the primary and metastatic tumors (early metastasis) with the metastatic tumors remaining closer to the normal copy number levels. Two patients had more intermediate levels of differences with one case (OV08-2) showing fewer differences to the baseline than the metastatic tumor. Whether tumor progression is specific to the patient, or simply the metastatic lesions that were randomly biopsied remains to be investigated. These significant differences in tumor progression timelines may be critical to better understanding the role of any given difference between primary and metastatic tumors.

**Figure 5:**
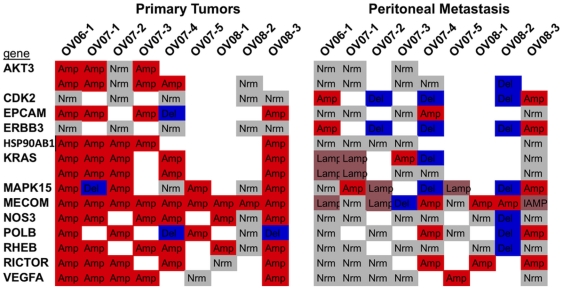
Genes identified as frequently amplified in primary ovarian tumors by the TCGA. Genes frequently amplified in primary tumors were suggested by the TCGA as potential chemotherapeutic targets. While primary tumors we studied agreed well with the TCGA findings, we find that metastatic, and likely residual, tumors do not regularly share the same amplifications. This should be considered prior to chemotherapeutic recommendations. Amp: amplified, Lamp: low-level amplification (not as amplified as in primary tumor) Nrm: no CNV with respect to HapMap baseline, Del: deletion.

With the recent comprehensive genomic analysis results published by the TCGA group on ovarian primary tumors we attempted to identify differences and similarities between metastatic tumors studied here and the TCGA findings. In their analysis they observed regular amplifications of certain genes that are already chemotherapeutic targets and suggested these for possible treatment options. As expected, many of our primary tumors contained amplifications of the same genes, however, many of these genes were not amplified in their matching peritoneal metastasis (Figure 5). Indeed, among the 9 patients' primary tumors we observed 64 amplification events in 13 genes identified by the TCGA as regularly amplified. Only 6 (9%) of the events were as highly amplified in the matched metastases and altogether only 15 (23%) had any form of amplification in the metastases. This observation is important as the typical complications of ovarian cancer result from peritoneal recurrence after surgical removal of the primary tumor. These results suggest that chemotherapy treatment recommendations made only on amplifications observed in the primary tumor may not be effective due to the residual metastatic disease not sharing the same amplifications.

Importantly, by using matched primary and metastatic lesions we could detect regions that had continued to change after the metastatic spread. These can be identified as amplifications and deletions that are shared between both primary and metastatic tumors when compared to a normal baseline and are further annotated as amplifications and deletions when the matched tumors are compared to each other (Table S6). For example, the chromosome 8q region is amplified in both primary and metastatic tumors (Figure 1a, Figure 1b). However, because the primary tumor is further amplified over matched metastatic tumors in at least 3 patients, the region is documented as a peritoneal metastatic deletion when the primary is used as a baseline (Figure 3). For consistency, we focused our analysis on the Cytokine signaling pathway (Table S6). In the case of shared amplifications that further amplify in the primary tumor we observed genes such as EGFR, GHR, IL7 and TNFR ligands among others. Genes that were amplified in both and further amplified in the metastatic tumor were almost exclusively IFNA related and were in the IFNA cluster on Chromosome 9p (Table S6). Indeed we observed continuing amplification in the metastasis for IFNA 1,2,4–8,10,13,14,16, 17 and 21. Shared deletions contained genes that continued to amplify in the peritoneum and these included FIGF (VEGF-D), IL 3,4,5,6ST,11, and 13 among others.

## Discussion

To our knowledge this is the first published report of comparison of genome-wide CNVs between matched primary and metastases in ovarian cancer. All in all our data agree well with previous CNV analysis of ovarian primary tumors and this supports the new findings in the matched peritoneal metastasis [9], [11]. Analysis of CNV regions shared among tumor types showed affected genes involved in numerous cancer pathways including the amplifications in the JAK/STAT pathway and deletions in the Apoptosis pathways. The role of JAK/STAT pathway has already been suggested in ovarian cancer. Indeed Colomiere *et al.* suggested a cross talk between EGFR and Il6Receptor in the EMT transition in ovarian carcinoma. They demonstrated the role of STAT3 in IL6 mediated migration of the cancer cells through EMT [21]. Seo et al. showed that LPA, which plays a primordial role in the occurrence of metastatic lesions in ovarian cancer, also activates STAT3 through secretion of IL6 and IL8 [22]. Meinhold-Heerlein et al. demonstrated that G2/G3 cancers were characterized by the expression of genes associated with the cell cycle and by STAT-1-, STAT-3/JAK-1/2-induced gene expression [23]. In this study we have observed that the role of JAK/STAT pathway changes may begin very early in tumor progression as many CNVs in this pathway were shared between primary and metastatic tumors. This would suggest that this pathway might be as important as other very early genetic abnormalities in tumor progression such as TP53 mutations. Changes in the pathway may then be specifically selected for by metastatic environmental requirements.

We further identified pathways that were affected in primary or metastatic tumors specifically. We observed primary tumor deletions in multiple CC chemokine genes (ligand or receptors) in all but one patient (OV08-2) suggesting this pathway is altered with high frequency. While we realize the number of cases is too small to draw firm conclusions, the relative homogeneity of ovarian cancer CNVs (our data as well as Bowtell group) might advocate for the possibility of only a few pathways being required in the change toward metastasis. Genome-wide CNV analysis on a greater number of patients will allow us to determine if indeed the pathways to metastasis are few and therefore consistent. Interestingly, patient OV08-2 was the only patient with refractory cancer in our cohort. Whether the complete absence of CC chemokine deletions in the primary tumor is related to this refractory state will require further investigation of a higher number of such patients. While the approach of using matched primary and metastatic lesions to study ovarian cancer genome-wide CNVs is unique in this study, a similar study in breast cancer has been reported [16]. Similar trends in the scale of differences between primary tumors and metastases were observed. By using the SNP6.0 array we increased resolution significantly allowing more refined determination of CNV segment boundaries. This increase in resolution highlighted the numerous differences between primary tumors and their metastases.

It is clear from our study that metastatic tumors are different from their ovarian primary source. Microenvironment pressures as well as the requirement for migration may select for copy number variations in these different pathways. The most frequently differentiating pathway we observed among primary and metastatic tumors was in the cytokine family of genes. This finding was corroborated by similar trends in gene expression data. The frequent involvement of cytokines in immune response and migration in cancer makes this an interesting finding. Indeed the role of immune infiltration has been recently described in different tumors including colon cancer and ovarian cancer [24]–[26]. More targeted studies are required in order to understand the differential immune environment in the primary and metastatic lesions. The understanding of the subtle microenvironment differences might allow the modulation of the immune response in order to avoid peritoneal recurrences [26].

Recently the TCGA group published the results of their comprehensive analysis of 489 patients with high-grade serous ovarian adenocarcinoma [10]. While the information on primary tumors from the TCGA is critical, we have shown that many targets of chemotherapy that are regularly amplified in both the TCGA and our samples are not amplified in matched metastatic tumors. Treatment decisions will need to carefully consider the genomic differences between primary and residual/metastatic tumors prior to chemotherapeutic recommendation.

Our study highlights the benefit and importance of performing paired analysis of primary tumors and their metastatic lesions in ovarian cancer. While comparison of primary and metastasis as groups provided insight into cancer development, the matched analysis allowed more specific detection of consistent differences. Indeed advanced disease allows access to not only the primary but also the different metastatic sites. It has been clearly demonstrated that the patients' prognosis relies on tumor residue; therefore it is critical to understand the biology of the metastatic lesions in order to design appropriate new therapeutic approaches. The results presented here should be a step in that direction.

## Materials and Methods

### Ethics Statement

All the samples were collected in the department of Gynecologic Oncology at the institut Claudius Regaud (DQ, AR). The project was reviewed and approved by the institution's Human research Ethics Committee. All patients included in the study gave informed written consent prior to surgery. 9 patients with advance Stage III or IV papillary serous ovarian adenocarcinoma were prospectively enrolled in this study at the time of primary surgery before any treatment was given. The patients had a biopsy of the primary lesion as well as a peritoneal metastasis outside of the pelvis. In order to ensure very little contamination by the stromal components the biopsies specifically took the tumoral nodules without the underlying peritoneal elements. All biopsies were immediately liquid nitrogen snap frozen. A representative haematoxylin and eosin stained section was assessed and samples with 80% epithelial cells and less than 20% of necrosis (criteria used by the TCGA group [10]) were used for DNA and RNA extraction from the whole tissue.

### RNA and DNA isolation

DNA and RNA were isolated using QIA-cube technology as per the manufacturer instructions.

### Affymetrix SNP Array 6.0 Processing

We used the Affymetrix Genome-Wide SNP Array 6.0 for the genomic analysis for the detection of copy number changes in this study. The workflow of Affymetrix Genome-Wide SNP Array 6.0 strictly followed the cytogenetic protocol from the manufacturer. 250 ng of total genomic DNA have been analyzed. The normal controls will be obtained from the 270 HapMap samples provided by Affymetrix.

### Quantitative-PCR Validation of Copy Number Variations

We selected a subset of regions identified as varying in copy number between primary tumor and peritoneal metastasis. As endogenous controls, we selected 3 gene regions that were shown by array analysis to not be amplified or deleted in our samples. Primers were designed using Primer3Plus on the hg19 version of the human genome (Table S1). For each primer pair quantitative PCR (QPCR) was conducted in triplicate on an Applied Biosystems 9700 Real-Time PCR machine using a 10 ul reaction of KAPA SYBR FAST Universal 2× qPCR Master Mix (Kapa Biosystems), 1.25 pmol each primer and 5 ng of genomic DNA and cycled according to the manufacturers recommended protocol. Analysis was conducted with the Applied Biosystems Relative Quantitation Manager software to calculate delta-delta Ct. Sample were normalized to the endogenous controls and peritoneal metastasis results were checked using the primary tumor samples as baseline reference.

### Data analysis

#### SNP arrays (Copy Number Variation analysis)

Data from the SNP6.0 arrays were analyzed using the PARTEK Genomics Suite software with recommended normalization settings. Each sample was compared to a HapMap distributed baseline to identify amplified and deleted regions using as segmentation algorithm within PARTEK. Segments showing copy number variation were only reported if they occurred in at least 3 patients with an individual patient False Discovery Rate (FDR) no greater than 20%.

#### Functional Analysis

Gene lists from both the gene expression and copy number variation analysis were entered into DAVID [13] and KEGG pathways enriched with Benjamini-Hochberg score of less than 0.25 were selected.

## Supporting Information

Table S1 **Primers used for qPCR validation of CNVs.** Primers including controls, their product coordinates on hg19 and their sequence are provided.
(XLSX)

Table S2 **Quantitative-PCR validation results of CNVs.** 11 regions in 5 genes were used to determine copy number in regions identified by arrays as deviating from 2 copies. Non-concordant results are highlighted in red.
(XLSX)

Table S3 **Primary ovarian tumor copy number variation regions identified in 9 patients.** Segments of variation including chromosomal location, which patients are amplified and deleted, and genes within the region are listed.
(XLSX)

Table S4 **Peritoneal metastasis tumor copy number variation regions identified in 9 patients.** Segments of variation including chromosomal location, which patients are amplified and deleted, and genes within the region are listed.
(XLSX)

Table S5 **Genes identified in copy number variation regions both shared by primary and metastatic tumors and those specific to each tumor type.** Both official gene symbols and Refseq IDs are provided. Comparisons include using a HapMap provided baseline (normal) or comparing the Peritoneal metastasis to the primary tumor baseline (Peri V Primary).
(XLSX)

Table S6 **Cytokine-Cytokine Receptor gene Copy Number Variations.** Cytokine/Receptor genes were noted for presence in CNVs for all tumor comparisons conducted. Amplifications are colored in red and deletions in blue. The CC subfamily is especially deleted in primary but not metastatic tumors.
(XLSX)

Table S7 **Peritoneal metastasis tumor copy number variation regions when compared to matched primary ovarian tumors identified in 9 patients.** Segments of variation including chromosomal location, which patients are amplified and deleted, and genes within the region are listed.
(XLSX)
